# Modeling diseases of aging in larval zebrafish, a paradoxical yet powerful strategy

**DOI:** 10.1093/genetics/iyag027

**Published:** 2026-03-10

**Authors:** Güliz Gürel Özcan, Jason Rihel

**Affiliations:** Department of Cell and Developmental Biology, University College London, London WC1E 6BT, United Kingdom; Department of Cell and Developmental Biology, University College London, London WC1E 6BT, United Kingdom

**Keywords:** neurodegeneration, Alzheimer's disease, Parkinson's disease, ALS, zebrafish, genetic disease modeling, behavioral fingerprinting, brain-wide activity mapping, behavioral pharmacology

## Abstract

Neurodegenerative diseases are a set of devastating medical conditions in which neuronal loss associated with the aggregation of toxic proteins leads to progressive cognitive impairment. These diseases are usually modeled in animals by mimicking late disease stages through genetic modifications that aggressively accumulate proteins that damage the brain. However, these diseases typically unfold over decades, and disease-associated genes are known to have important, but understudied, biological functions in early life stages. To address this research gap, we suggest that the larval zebrafish, which has conserved orthologs of most neurodegeneration-linked genes, is an excellent model to examine early mechanisms that set the stage for disease progression, such as altered neuronal function, synaptic re-wiring, and proteostasis. We propose a systematic genetic modeling and phenotyping pipeline in zebrafish that integrates CRISPR editing, high-throughput behavioral assays, brain-wide activity mapping, and pharmacological screens to capture neurodegenerative disease-related changes that occur well before clinical disease emerges. Studying diseases of aging in larval zebrafish may sound paradoxical; however, by uncovering cellular dysfunction at the earliest stages of disease in a living vertebrate brain, this approach could identify critical therapeutic targets at timepoints before degeneration becomes irreversible.

## Introduction

Neurodegenerative diseases are progressive and devastating conditions characterized by the gradual loss of synapses and neurons, leading to cognitive decline, motor deficits, and other neurological impairments. Typically associated with aging, the prevalence of these conditions worldwide is growing as many societies shift to older populations. For example, Alzheimer's disease (AD), the most common form of dementia, currently affects over 32 million people worldwide and is expected to rise to nearly 119 million by 2050 (Nichols et al. 2022). More broadly, 57 million people globally were living with dementia in 2019, a figure expected to triple to 153 million by 2050 (Nichols et al. 2022). To combat this immense human and financial toll, we need effective long-term treatments, but these mostly remain elusive. Even in cases where we have modestly successful therapies, such as L-DOPA for managing motor symptoms in Parkinson's disease (PD), these do not slow or halt disease progression and become ineffective over time (Olanow et al. 2009).

One of the major challenges to developing effective treatments for neurodegenerative disease is the difficulty in studying the molecular and cellular mechanisms that underpin the development and progression of these diseases in the lab. Despite variations in symptoms and affected brain regions, many neurodegenerative disorders share the aggregation or gain of function of toxic misfolding proteins as a core pathological feature. For example, AD is associated with the aggregation of amyloid-beta (Aβ) and hyperphosphorylated tau; PD is defined by misfolded α-synuclein; Amyotrophic lateral sclerosis (ALS) and frontotemporal dementia (FTD) are characterized by TDP-43 and C9ORF72 aggregates; and prion diseases are triggered by the misfolding of Prion proteins. These toxic aggregates can impair various cellular processes, such as proteostasis and mitochondrial function, and can induce neuroinflammation to trigger additional pathological pathways, with a cumulative effect that ultimately leads to neuronal dysfunction and cell death. Since these toxic proteins build up over years or decades, are not composed of homogenous species, and have distinct progressive effects on brain physiology over a long time-course, modeling the formation and accumulation of toxic aggregates in animal models poses considerable experimental challenges.

Given the central role of toxic protein accumulation, most animal models focus on gain-of-function approaches, using genetic modifications to over-express human disease genes or introduce disease-associated gain-of-function mutations. These models are essential tools for understanding late-stage disease mechanisms and testing potential therapies. However, these models are not without their limitations. One major issue is that these models necessarily seek to hyper-accelerate disease progression to capture late-stage pathology in short-lived animals, overlooking early molecular and cellular changes that may be crucial for understanding disease initiation. For example, transgenic mice that over-express mutant *APP* or *PSEN1/2* are used to rapidly accumulate amyloid plaques (Oakley et al. 2006; Radde et al. 2006), and PD models frequently over-express α-synuclein to quickly produce Lewy body-like inclusions and dopaminergic neuron loss (Feany and Bender 2000; Richfield et al. 2002). While these models are capable of reproducing the toxic protein burden of advanced disease, they tend to obscure earlier processes such as synaptic dysfunction, mitochondrial impairment, neuroinflammation, and disrupted developmental programs that may be equally important for disease initiation (Wilson et al. 2023). Ultimately, preventing disease progression will likely require reversing processes that may have irrevocably initiated decades earlier.

Another significant challenge for these traditional models lies in addressing the broad and expanding landscape of genetic risk factors uncovered by recent genome-wide association studies (GWAS) that have identified numerous novel loci associated with increased risk for neurodegenerative diseases. For example, in AD, variants in genes like *APOE* and *TREM2* have long been implicated in amyloid clearance and immune responses (Holtzman et al. 2000; Jonsson et al. 2013), but a 2022 GWAS meta-analysis greatly expanded the risk-variants to include 75 loci associated with late-onset AD, highlighting pathways involved in amyloid processing, immune signaling, and lipid metabolism (Bellenguez et al. 2022). In ALS, only a handful of genes, such as *UNC13A* and *C9ORF72* (Webster et al. 2016; Willemse et al. 2023), were established risk loci, yet methods that combine epigenetic profiling with GWAS data detected nearly 700 additional loci that confer heritable risk (Zhang et al. 2022).

The practical time and cost constraints of traditional rodent models make them poorly suited for the types of rapid screening and whole genome-based approaches that are required to explore the basic functions of genes newly linked to neurodegenerative disease. The zebrafish is one such model that provides a complementary system that directly addresses these gaps. Their optical transparency enables rapid, in vivo readouts of brain function during critical early stages; their short generation time and high fecundity support large-scale genetic screens; CRISPR-based mutagenesis allows efficient manipulation of candidate genes; and small molecules are easily absorbed from the water to affect the brain and other tissues. Together, these features make zebrafish uniquely suited for exploring how diverse genetic loci influence early neural development, circuit function, and vulnerability to later degeneration.

### Zebrafish as a model for neurodegenerative disease

Zebrafish have long served as a valuable model organism in developmental biology due to their external fertilization and rapid progression from a single cell to a free-swimming larva in less than 5 d. These features enable real-time imaging of cellular and molecular events and support efficient genetic manipulation for mapping genetic and cellular interactions during early embryogenesis. More recently, neuroscientists have harnessed zebrafish strengths such as the ability to perform high-throughput behavioral assays, non-invasive in vivo imaging, and neuronal manipulations via optogenetic control to decode circuit-level mechanisms underlying behaviors like hunting and sleep (Doszyn et al. 2024).

These features also make zebrafish an attractive system for modeling human diseases. The evolutionary conservation of genes between zebrafish and humans, with orthologs for over 82% of human disease-related genes and approximately 70% similarity on average per protein (Howe et al. 2013), enables modeling of most human diseases in zebrafish, particularly those with developmental origins such as autism, epilepsy, and cancer (Stewart et al. 2014; Adhish and Manjubala 2023). Especially potent is the ability to use these disease models in medium- and high-throughput drug screens in which the compounds are placed directly in the water and efficiently absorbed by the larvae (He et al. 2013). Such small molecule screens have now identified nearly a dozen disease modifying compounds that have progressed to human clinical trials, such as clemizole, a serotonin 5-HT_2_B receptor modulator identified as a seizure suppressor in zebrafish models of Dravet syndrome that is now in Phase 3 trials (Griffin et al. 2019).

Using zebrafish to investigate neurodegenerative disease may seem paradoxical at first glance, given that neurodegenerative diseases manifest as a consequence of aging, while many advantages of zebrafish rely on tools and methodologies only routinely available in very young animals. Like mice, zebrafish live around 2 yr and do not spontaneously develop hallmark pathologies such as amyloid plaques or Lewy bodies, which means that modeling late-stage disease in adult zebrafish requires the same aggressive overexpression strategies used in rodent models. Moreover, adult zebrafish exhibit significant regenerative capacity (Kizil et al. 2012) and lifelong neurogenesis in major brain areas like the forebrain (Chouly and Bally-Cuif 2024), which contrasts sharply with the limited regenerative potential of the mammalian brain (Sorrells et al. 2018).

Instead of a focus on late stages of disease, we argue that larval zebrafish offer a unique experimental paradigm for studying the earliest stages of neurodegenerative disease initiation and progression. Their high-throughput potential makes the zebrafish larva particularly well suited to address the expanding set of neurodegenerative disease risk factors uncovered by large-scale human genomic studies. Modern gene editing tools like CRISPR/Cas9 accelerate the study of basic functions of genes by enabling rapid generation and phenotyping of loss- and gain-of-function mutations in neurodegenerative disease-associated genes, even within the same edited generation (Kroll et al. 2025). Live imaging of cellular and behavioral responses to acute exposure to disease proteins provides insights into the early perturbations caused by these toxic species that may lead to longer-term disease progression. For example, studies injecting a variety of Aβ oligomeric species into zebrafish have revealed unexpected diversity in the genetic pathways by which Aβ modulates behaviors like sleep (Ozcan et al. 2020, 2024). Such models can also be rapidly combined with small molecule or genetic interventions to identify candidate therapeutics; for example, Cas13-mediated targeting of toxic *C9orf72* expansions improved motor function in a zebrafish overexpression model of ALS (Kempthorne et al. 2025).

Together, these advantages constitute a versatile experimental pipeline to study neurodegenerative disease in larval zebrafish, beginning with genetic manipulation of disease risk genes and extending to behavioral phenotyping and therapeutic drug screening. These approaches are now being applied to a variety of neurodegenerative diseases, including AD, PD, ALS, and prion disorders.

### Neurodegenerative disease genes and zebrafish models

We have already highlighted the high genetic conservation between humans and zebrafish for genetic disease. Neurodegenerative diseases are no exception, with clear orthologs in zebrafish for most disease risk genes (Table 1). In this section, we discuss the many zebrafish genetic models that have already been established across major classes of neurodegenerative disease.

**Table 1.** Conserved neurodegenerative disease risk genes.

Disease	Human gene full name	Abbreviation	Zebrafish ortholog	Protein identity (%)	Reference
AD	A disintegrin and metalloproteinase 10	ADAM10	*adam10b*	72.5	Kroll et al. (2025)
AD	Anterior Pharynx Defective 1 Homolog B	APH1B	*aph1b*	62.8	Kroll et al. (2025)
AD	Apolipoprotein E	APOE	*apoea, apoeb*	22.4, 25.5	Babin et al. (1997), Durliat et al. (2000), Kroll et al. (2025)
AD	Amyloid Beta Precursor Protein	APP	*appa, appb*	68.6, 68.2	Kroll et al. (2025)
AD	Bridging Integrator 1	BIN1	*bin1a, bin1b*	53.2, 63.7	Kroll et al. (2025)
AD	Sortilin-related receptor	SORL1	*sorl1*	63.5	Kroll et al. (2025)
AD	Amyloid Beta Precursor Protein	APP	*appa, appb*	80, 71	Musa et al. (2001), Ozcan et al. (2024)
AD	Presenilin 1	PSEN1	*psen1*	73.9	Leimer et al. (1999)
AD	Presenilin 2	PSEN2	*psen2*	74	Groth et al. (2002)
AD	Presenilin Enhancer Protein 2	PEN2	*pen2*	74	Groth et al. (2002)
AD	Anterior Pharynx Defective 1 Homolog A & B	APH1A&B	*aph1*	62	Francis et al. (2002)
AD	Nicastrin	NICASTRIN	*nicastrin*	56	Lim et al. (2015)
AD	Microtubule-Associated Protein Tau	TAU	*mapta, maptb*	58, 62^a^	Chen et al. (2009)
PD	Alpha-synuclein	SCNA	*sncga, sncgb, sncb*	N/A	Sun and Gitler (2008), Milanese et al. (2012)
PD	Parkin	PARKIN	*parkin*	62	Flinn et al. (2009)
PD	DJ-1	DJ-1	*dj-1*	83	Bai et al. (2006)
PD	Leucine-Rich Repeat Kinase 2	LRRK2	*lrrk2*	48	Suzzi et al. (2021)
PD	PTEN Induced Kinase 1	PINK1	*pink1*	54	Anichtchik et al. (2008)
HD	Huntingtin	HTT	*htt*	70	Karlovich et al. (1998)
ALS	TAR DNA Binding Protein 43	TARDBP (TDP-43)	*tardbpc*	73	Oliveira et al. (2023)
ALS	C9orf72	C9ORF72	*zgc:100846*	73	Oliveira et al. (2023)
ALS	Superoxide Dismutase 1	SOD1	*sod1*	70	Oliveira et al. (2023)
ALS	Fused in Sarcoma	FUS	*fus*	64	Oliveira et al. (2023)
Prion	Prion Protein	PRNP	*prp1, prp2*	25, 33^a^	Cotto et al. (2005)
SCA	Ataxin 1	ATXN1	*atxn1a, atxn1b*	46, 37	Carlson et al. (2009)
SCA	Ataxin 2	ATXN2	*atxn2*	62	Vauti et al. (2025)
SCA	Ataxin 3	ATXN3	*atxn3*	85^b^, 52^a^	Felicio et al. (2024)
SCA	Potassium Voltage-Gated Channel Subfamily C Member 3	KCNC3	*kcnc3a, kcnc3b*	66, 67	Mock et al. (2010), this study
SCA	Calcium voltage-gated channel subunit α1 A	CACNA1A	*cacna1aa*, *cacna1ab*	72, 71	Gawel et al. (2020)

^a^Within the conserved C-terminal region.

^b^Within the N-terminal Josephin Domain.

#### Alzheimer's disease

AD is a progressive neurodegenerative disorder characterized by the accumulation of amyloid-beta (Aβ) plaques and tau neurofibrillary tangles and is the leading cause of dementia. Familial AD (FAD) accounts for 4% to 8% of all AD cases (Chapman et al. 2001), which are caused by autosomal dominant mutations in either amyloid precursor protein (APP) (Chartier-Harlin et al. 1991; Murrell et al. 1991), presenilin-1 (PSEN1) (Campion et al. 1995; Cruts et al. 1995), or presenilin-2 (PSEN2) (Sherrington et al. 1996). APP is cleaved by a gamma-secretase made up of the Presenilins to generate Aβ, which aggregates in the brains of AD patients and is considered a major driver of disease progression (Murphy and Levine 2010).

The zebrafish model is a well-suited tool for investigating these pathways, with conserved genes and developmental processes, including the machinery to generate Aβ species associated with AD (Table 1). These zebrafish studies highlight the conservation of AD-relevant pathways and show how unique phenotypes such as disrupted sleep or altered myelination can be rapidly and effectively modeled.

##### APP and PSEN

Zebrafish possess 2 homologues of amyloid precursor protein (APP), *appa* and *appb*, each sharing over 70% identity with human APP-695, APP751, and APP770, which are the most common isoforms (Musa et al. 2001). Despite having 2 APP orthologs due to an ancient genome duplication event (Glasauer and Neuhauss 2014), Appa and Appb have partially non-overlapping expression domains in the zebrafish brain and resemble distinct human APP isoforms, with Appa more similar to KPI containing APP751 and APP770, while *Appb* is more similar to APP695 (Musa et al. 2001; Ozcan et al. 2024). Thus, the tissue expression and isoform-specific functions of mammalian APP, which derive from a single gene, are likely fulfilled in zebrafish by partially non-redundant Appa and Appb, a common feature of duplicated genes (Force et al. 1999). Zebrafish also express a functional γ-secretase complex that cleaves APP to generate Aβ peptides. Both Aβ40 and Aβ42 are detectable in zebrafish but are abrogated in either *appa* or *appb* mutants and completely abolished by the γ-secretase inhibitors or by mutations in *presenilin-1* (Ozcan et al. 2024; Kroll et al. 2025), demonstrating conserved Aβ production. The proportion of Aβ42 (5% to 10% of total Aβ) to Aβ40 (Ozcan et al. 2024) is comparable to the ratio detected in mammals (Murphy and Levine 2010; Hansson et al. 2019), suggesting a tight regulation of Aβ production and clearance in zebrafish that is similar to other species.

Genetic loss-of-function studies of APP and Presenilins in zebrafish have revealed numerous roles for these genes in a variety of biological processes, from development to neural function and behavior. Mutants that lack *appa* have been reported to have increased susceptibility to seizures (Kanyo et al. 2020), while *appb* mutants have altered cell adhesion that affects but does not prevent early developmental events (Banote et al. 2020). Double mutants that lack both *appa and appb* have altered sleep, a phenotype highly relevant for AD progression, with *appb* knockout zebrafish in particular displaying fragmented nighttime sleep that resembles early sleep disturbances in AD (Ozcan et al. 2024; Kroll et al. 2025). In *psen1^−/−^* mutants, histaminergic neurotransmission is altered, a finding relevant to AD since histamine modulates wakefulness and cognitive function (Sundvik et al. 2013). Zebrafish *psen2* F0 knockouts generated by Crispr/Cas9 injection also have altered sleep (Kroll et al. 2025). Moreover, mutants lacking *psen2* have reduced ER–mitochondria contacts and increased basal autophagy in neurons (Barazzuol et al. 2023), both processes implicated in AD pathogenesis. Together, these studies underscore how *appa/b*, *psen1*, and *psen2* influence cellular pathways essential for neural homeostasis and how zebrafish can reveal early consequences of alterations in AD-related genes.

Zebrafish have also been used in AD gain-of-function studies, through both genetic misexpression of APP and exposure to exogenous Aβ. Misexpression of a mutant form of human APP in zebrafish led to some signatures of cerebral amyloidosis and angiopathy (CAA) and neuron loss, hallmarks of AD (Pu et al. 2017). Injection of high concentrations of Aβ peptides impaired oligodendrocyte differentiation and spinal cord myelination via disrupted PKC signaling (Balantzategi et al. 2025) and also altered neurogenesis by inducing inflammatory responses (Bhattarai et al. 2017). More recent studies that have carefully controlled the oligomeric species and concentrations of Aβ demonstrated that different Aβ oligomers, including the shortened P3 peptides (Aβ17-42) that are also observed in human AD plaques, can either increase or decrease sleep via distinct signaling cascades (Ozcan et al. 2020, 2024), thus linking Aβ toxicity to early behavioral phenotypes relevant to AD.

##### Tau/MAPT

Mutations in the *microtubule-associated protein tau* (*MAPT*, *TAU*) gene are a known risk factor for AD and other tauopathies (Bird et al. 1997; Hutton et al. 1998). In AD, tau becomes hyperphosphorylated and aggregates into neurofibrillary tangles that are believed to disrupt neural function and contribute to neurodegeneration (Grundke-Iqbal et al. 1986). Zebrafish models have been developed to explore both early and late tau-related pathology, particularly through transgenic overexpression of human Tau. Expression of fluorescently tagged human Tau variants in zebrafish led to delayed neuronal differentiation, behavioral deficits, and AD–like pathology, including cytoskeletal disruption and Tau hyperphosphorylation (Tomasiewicz et al. 2002; Paquet et al. 2009). Similarly, expressing the AD human risk *A152T tau* allele in zebrafish caused neurodegeneration in the retina and spinal cord (Lopez et al. 2017). This neurodegeneration could be ameliorated by induction of autophagy, highlighting the model's suitability for identifying disease modifiers and candidate therapeutic targets. However, direct misexpression of the human *TAU P301L* mutation in radial glia or neurons led to Tau hyperphosphorylation in adult zebrafish brains but notably did not result in neurofibrillary tangle formation or extensive neurodegeneration (Cosacak et al. 2017). This finding suggests that zebrafish may possess intrinsic protective mechanisms against Tau aggregation and toxicity, an important contrast to mammalian models and a potential area for therapeutic exploration.

##### APOE

The apolipoprotein E (*APOE*) gene is one of the strongest genetic risk factors for late-onset AD (Corder et al. 1993). Among its alleles, the ε4 variant is particularly detrimental, as it impairs Aβ clearance and compromises blood-brain barrier integrity (Deane et al. 2008). Zebrafish possess 2 isoforms of ApoE (*apoea* and *apoeb*) which are expressed in distinct tissues (Kroll et al. 2025), and functional studies have begun to uncover how these isoforms contribute to neural regulation. For example, Crispr F0 knockouts of *apoeb* in zebrafish display disrupted sleep patterns (Kroll et al. 2025) that mirror the sleep fragmentation seen in early AD (Blackman et al. 2022). In a separate line of research, exposure of zebrafish to an amino-terminal fragment of the human APOE4 protein caused increased PHF-1 immunoreactivity, which is a marker of pathological tau phosphorylation, behavioral abnormalities, and elevated mortality (Mccarthy et al. 2022). These findings suggest that APOE4 toxicity extends beyond Aβ clearance deficits and may directly contribute to Tau pathology.

#### Parkinson's disease

PD is the second most prevalent progressive neurodegenerative disorder that affects over 11.77 million people as of 2021 (Driver et al. 2009; Luo et al. 2025). The disease primarily affects individuals in older ages, with clinical symptoms, including tremors, bradykinesia, and motor impairments (Sveinbjornsdottir 2016). Pathological hallmarks of PD include the presence of Lewy bodies and Lewy neurites that contain aggregated α-synuclein, a presynaptic protein involved in regulation of neurotransmitter release via the interaction with synaptic vesicles (Snead and Eliezer 2014). Genetic mutations in α-synuclein lead to its aggregation, which then contributes to neurotoxicity (Conway et al. 2000). PD can also arise from mutations in other genes such as PARK2 (*Parkin*) (Kitada et al. 1998), PARK7 (*DJ-1*) (Hague et al. 2003; Macedo et al. 2003), PINK1 (PTEN-induced kinase 1) (Hofer and Gasser 2004), and LRRK2 (*Leucine-Rich Repeat Kinase 2*) (Zimprich et al. 2004). These mutations are believed to cause familial forms of PD through both shared and gene-specific mechanisms. For example, loss-of-function mutations in *PINK1* or *parkin* impair mitophagy and mitochondrial quality control, fostering oxidative stress and potentially facilitating α-synuclein pathology (Pickrell and Youle 2015). *DJ-1* deficiency compromises the cell's ability to mitigate oxidative stress and, under normal conditions, may act as a redox-sensitive chaperone that inhibits α-synuclein aggregation (Shendelman et al. 2004). In contrast, pathogenic *LRRK2* mutations (eg G2019S) promote α-synuclein aggregation and propagation and also drive neuroinflammation and mitochondrial dysfunction (Dues and Moore 2020). These pathogenic processes converge most strongly in dopaminergic neurons of the substantia nigra, which are especially vulnerable due to their high metabolic demand and reliance on mitochondrial integrity. As a result, mutations in these genes ultimately promote the progressive dopaminergic cell loss that defines PD pathology (Surmeier 2018).

Zebrafish have a well characterized dopaminergic system and highly conserved genes linked to PD (Table 1).

#### SCNA

α-Synuclein aggregation is a hallmark of PD pathogenesis. While zebrafish lack a direct ortholog of human α-synuclein, they express 3 related synucleins, *sncga*, *sncgb*, and *sncb*, which share sequence similarity with human α-synuclein (Sun and Gitler 2008) but lack the conserved 12-residue hydrophobic motif that distinguishes human α-synuclein (Table 1). Zebrafish deficient in both β- and γ1-synucleins exhibit pronounced hypokinesia, delayed differentiation of dopaminergic neurons, and reduced dopamine levels compared to single knockouts (Milanese et al. 2012). Notably, these deficits could be rescued by expression of human α-synuclein, indicating that zebrafish synucleins have evolutionarily conserved roles in regulating dopamine homeostasis and motor behavior (Milanese et al. 2012).

Several zebrafish overexpression models have been developed to study human α-synuclein toxicity and PD-related pathology. Transgenic zebrafish expressing either wild-type or a mutant form human α-synuclein (*α-SYN A53T*) recapitulate key features of PD, including protein aggregation and neurodegeneration, and offer a model to investigate α-synuclein clearance kinetics and mechanisms of neuroprotection (Lopez et al. 2022). A zebrafish model that expresses human α-synuclein fused to GFP developed aggregates at presynaptic terminals independently of phosphorylation at serine-129, suggesting that this post-translational modification may not be essential for aggregation in vivo (Weston et al. 2021). Additional overexpression studies have shown that α-synuclein amplifies oxidative stress by increasing cytoplasmic peroxide flux, particularly when combined with mitochondrial inhibitors, thereby heightening dopaminergic neuron vulnerability (Van Laar et al. 2020). Another zebrafish model of α-synuclein toxicity had axonal degeneration that could be mitigated by upregulating PGC-1α, a regulator of mitochondrial biogenesis (O’donnell et al. 2014). Thus, zebrafish models of synucleinopathies mimic key aspects of PD and can be leveraged to identify potential therapeutic strategies.

#### PARK7

Mutations in the DJ-1 protein, encoded by the *PARK7* gene, cause autosomal recessive forms of PD (Bonifati et al. 2003). In humans, DJ-1 is thought to function primarily as a protector against oxidative stress (Kahle et al. 2009). The zebrafish DJ-1 protein shares over 80% similarity with its human and mouse counterparts and is expressed widely throughout the body (Bretaud et al. 2007). Early studies using morpholino-based knockdown of *dj-1* in zebrafish reported no significant effect on dopaminergic neuron numbers under baseline conditions but found increased oxidative stress as indicated by elevated SOD1 levels (Bretaud et al. 2007). Consistent with this, challenging *dj-1* knockdown animals with hydrogen peroxide resulted in dopaminergic neuron loss, suggesting that DJ-1 plays a role in neuron survival particularly under stress or damage conditions (Bretaud et al. 2007). More recently, a CRISPR/Cas9-generated *dj-1* knockout showed age-dependent retinal degeneration (Gharbi et al. 2021) and reduced tyrosine hydroxylase expression in older fish (Edson et al. 2019), supporting a conserved function of DJ-1 in maintaining neuron integrity.

#### PARKIN

Mutations in the *parkin* gene (*PARK2*) are responsible for autosomal recessive juvenile-onset PD (Kitada et al. 1998). Zebrafish Parkin shares 62% sequence similarity to human PARKIN (Table 1, Flinn et al. 2009). Morpholino knockdown of *parkin* in zebrafish led to a significant reduction in the number of dopaminergic neurons in the posterior tuberculum, a region analogous to the human substantia nigra (Flinn et al. 2009). Parkin knockdown also led to a specific reduction in mitochondrial respiratory chain complex I activity, representing the first vertebrate model to exhibit both a key pathogenic mechanism, complex I deficiency, and the hallmark feature of dopaminergic neuron loss that was observed in human patients with *parkin* mutations (Flinn et al. 2009). Validation of these morpholino results, which can have non-specific effects, awaits a *parkin* knockout model.

#### PINK1

Autosomal recessive mutations in the *PTEN-induced putative kinase 1* (*PINK1)* gene typically cause early-onset PD (Valente et al. 2004; Bonifati et al. 2005). *PINK1* is known to regulate mitophagy, mitochondrial function, and oxidative stress, but its precise role in PD remains unclear (Brown et al. 2021). Zebrafish Pink1 is 54% identical to the human PINK1 (Anichtchik et al. 2008). Unlike *Pink1* knockout mice, which show no dopaminergic neuron loss, *pink1*-mutant zebrafish exhibit impaired neurogenesis of dopaminergic neurons, leading to fewer dopaminergic neurons in both larvae and adults, and altered mitochondrial function and structure (Flinn et al. 2013; Brown et al. 2021). The *pink1* mutant zebrafish model has been used to identify pathways that can prevent dopamine neuron loss, including genetic and pharmacological inhibition of a mitochondrial calcium uniporter (Soman et al. 2017) as well as disruption of the apoptosis regulator, TigarB (Flinn et al. 2013), showing that zebrafish PD models can help discover candidate therapeutic pathways.

#### LRRK2

Mutations in the *LRRK2* gene are known genetic risk factors for both familial and sporadic PD (Zimprich et al. 2004; Gilks et al. 2005), potentially by promoting α-synuclein accumulation (Bae et al. 2018). Human LRRK2 and zebrafish Lrrk2 proteins share the same domains and 48% conservation overall (Suzzi et al. 2021). One study found that zebrafish *lrrk2* loss-of-function mutants had a weakened immune response to bacterial infection and were hyperactive as adults (Sheng et al. 2018). Another *lrrk2* mutant study found they had subtle brain phenotypes early in development, including a temporary reduction in catecholaminergic neurons, followed by a late-onset, progressive degradation of dopamine and serotonin levels that was linked to elevated monoamine oxidase activity (Suzzi et al. 2021).

### Huntington's disease

Huntington's disease (HD) is an autosomal-dominant neurodegenerative disorder caused by a CAG repeat expansion in the huntingtin gene (*HTT*), leading to a polyQ repeat in the Huntingtin protein. Disease progression is linked to the number of CAG repeats, which influences the age of onset, severity, and penetrance of the disease (Langbehn et al. 2010). HTT plays a critical role in regulating organelle transport within neurons (Caviston et al. 2007) by binding directly to dynein or indirectly to huntingtin-associated protein 1 (HAP1) to transport various organelles (Engelender et al. 1997). Mutant Huntingtin (mHTT) forms unstable aggregates that accumulate in intranuclear inclusions, a hallmark of HD pathology (Difiglia et al. 1997). Impaired proteostasis of mHTT leads to neuronal toxicity, particularly in GABAergic medium spiny neurons in the striatum, resulting in brain cell loss (Graveland et al. 1985).

#### HTT

The zebrafish *huntingtin* (*htt*) gene shares approximately 70% similarity with the human protein (Karlovich et al. 1998) (Table 1). In zebrafish, Htt is ubiquitously expressed in the brain and may be important for the formation of telencephalic progenitor cells and pre-placodal cells (Henshall et al. 2009). Suppression of Htt expression in zebrafish using morpholino antisense oligonucleotides led to extensive neuronal apoptosis due to reduced expression of brain-derived neurotrophic factor (Diekmann et al. 2009). Other studies have shown that injecting mRNA encoding the N-terminal fragment of Htt with varying lengths of polyQ repeats results in severe developmental abnormalities and apoptosis in zebrafish embryos within 24 h (Schiffer et al. 2007). These early studies using morpholino-based knockdown suggested that *huntingtin* loss in zebrafish causes severe embryonic defects; however, these phenotypes were not replicated in CRISPR/Cas9 knockout *htt* models, which resulted only in reduced fitness at later developmental stages (Sidik et al. 2020). This finding challenges the relevance of the developmental phenotypes uncovered by earlier morpholino studies and shows that more work is needed, especially on gain-of-function models replicating CAG expansion repeat forms of *huntingtin*, to fully realize the potential of studying HD in zebrafish.

### Amyotrophic lateral sclerosis

ALS, also known as motor neuron disease, is a rare, progressive neurodegenerative disorder that affects the motor system (Kiernan et al. 2011). With an incidence of 2 to 4 per 100,000, ALS is fatal and currently untreatable (Kiernan et al. 2011). ALS leads to the degeneration of motor neurons in the brain and spinal cord, resulting in muscle weakness, atrophy, speech and swallowing difficulties, and eventually paralysis and death (Loeffler et al. 2016). ALS is primarily sporadic (90% of cases), but about 10% are familial, linked to mutations in several genes (Mulder et al. 1986). Mutations in *Superoxide Dismutase 1* (*SOD1*), *TAR DNA-Binding Protein 43* (*TARDBP*), *Fused in Sarcoma / Translocated in Liposarcoma* (*FUS*/*TLS*), and *Chromosome 9 Open Reading Frame 72* (*C9ORF72*) account for up to 70% of familial cases (Hardiman et al. 2017). Zebrafish possess orthologs of these genes (Table 1), and zebrafish models have been generated for all 4 genes, in many cases successfully replicating key hallmarks of ALS.

#### SOD1

SOD1 is a primarily cytosolic superoxide detoxifying enzyme. The ALS-linked SOD1 mutations destabilize the protein structure and promote toxic mitochondrial aggregates through abnormal disulfide cross-linking (Didonato et al. 2003), mitochondrial dysfunction (Israelson et al. 2010), and pro-oxidant activity (Yamazaki et al. 2022). Zebrafish and human SOD1 are 76% identical, and introducing the ALS-linked mutation T70I into zebrafish SOD1 displays hallmark ALS phenotypes such as early neuromuscular junction defects, oxidative stress sensitivity, and adult-onset motor neuron disease (Da Costa et al. 2013). Additional validation of zebrafish SOD1 models of ALS comes from stable transgenic zebrafish lines. Zebrafish overexpressing the familial ALS-linked G93A-SOD1 or G93R-SOD1 mutations show progressive motor deficits, early loss of neuromuscular junctions, disrupted motor neuron innervation, neuronal stress, muscle atrophy, paralysis, and late-stage motor neuron loss, recapitulating key features of ALS pathology (Ramesh et al. 2010; Sakowski et al. 2012; Mcgown et al. 2013).

#### TARDBP

TARDBP (TDP-43) is a nuclear protein involved in axonal transport and is a major component of pathological aggregates found in motor neurons of ALS patients. Zebrafish TARDBP shares 72% amino acid identity with its human TARDBP ortholog (Shankaran et al. 2008). Zebrafish double loss-of-function mutants in *tardbp* and the related *tardbpl* had muscle degeneration, vessel pattern defects, and impaired spinal motor neuron outgrowth (Schmid et al. 2013). Gain of function zebrafish models also recapitulate features of ALS. For example, overexpression of the ALS-linked *TARDBP-A315T* mutation caused motor dysfunction and abnormal motor axon morphology (Kabashi et al. 2010), while transgenic lines expressing wild-type or G348C mutant human TDP-43 showed reduced locomotor activity after touch, moderate axonopathy, and premature axonal branching (Lissouba et al. 2018).

An interesting gain-of-function TDP-43 approach was developed in zebrafish that uses a light-sensitive variant of opTDP-43 that enables precise experimental control of TDP-43 aggregation in vivo (Asakawa et al. 2020). Short-term light exposure that induced reversible cytoplasmic mislocalization of TDP-43 already led to myofiber denervation and axonal outgrowth defects, while prolonged illumination induced pathological TDP-43 aggregation and motor impairments (Asakawa et al. 2020). An ALS-linked mutation in the intrinsically disordered region of TDP-43 worsened the toxicity of these light-induced aggregates, suggesting this genetic disruption is a driver of TDP-43 oligomerization and motor neuron pathology (Asakawa et al. 2020). Such studies demonstrate the power of using light-based manipulations in the translucent zebrafish larvae to control TDP-43 aggregation dynamics and functions in vivo, a methodology that could easily be extended to study other neurodegenerative diseases involving protein aggregation.

#### FUS/TLS

The *FUS/TLS* gene encodes a protein involved in RNA binding, and mutations in *FUS* are associated with ALS (Kwiatkowski et al. 2009; Vance et al. 2009). Zebrafish have a FUS ortholog that is 64% identical to the human form (Oliveira et al. 2023). A genetic deletion mutant of zebrafish *fus* showed impaired motor abilities, shortened motor neuron length, fragmented neuromuscular junctions, and reduced lifespan (Bourefis et al. 2020). Gain-of-function studies in zebrafish also confirm a conserved role for Fus in ALS-related pathways. Overexpression in zebrafish motor neurons of the human FUS-R495X, which is a truncated variant associated with ALS that leads to loss of nuclear localization and cytoplasmic accumulation (Bosco et al. 2010), resulted in cytoplasmic FUS aggregation and induction of oxidative stress, mirroring key aspects of ALS motor neuron pathology (Bosco et al. 2010).

#### C9ORF72

The *C9ORF72* gene carries intronic hexanucleotide-repeat expansions, which can be transcribed into toxic sense and antisense RNAs and translated into aggregation-prone dipeptide repeat proteins, causing neuronal toxicity (Dejesus-Hernandez et al. 2011; Shaw et al. 2018). Transgenic zebrafish lines expressing C9orf72 hexanucleotide-repeat expansions have motor deficits, muscle atrophy, motor neuron loss, and mortality in early adulthood (Shaw et al. 2018). Hyperactivity is a well characterized phenotype in C9orf72 repeat expansion models and has been observed in mice overexpressing 66 or 149 G4C2 repeats (Chew et al. 2015). A recent zebrafish model replicated this hyperactivity and then demonstrated that targeted cleaving of the toxic C9orf72 mRNAs using Cas13 can improve this locomotor deficit (Kempthorne et al. 2025). Thus, zebrafish can effectively model C9orf72-associated phenotypes and can be used for exploring therapeutic interventions.

### Prion diseases

Prion diseases, or transmissible spongiform encephalopathies, are rare, fatal neurodegenerative disorders caused by misfolded prion proteins leading to brain damage. One of the major human prion diseases is Creutzfeldt-Jakob disease, which can be sporadic, familial, or acquired through exposure to contaminated materials (Goldfarb et al. 1992; Collinge and Rossor 1996; Collinge 1999). Other human prion diseases include Gerstmann–Sträussler–Scheinker syndrome, which is characterized by adult onset of memory loss, dementia, and ataxia (Hsiao et al. 1989; Irie et al. 2024), and fatal familial insomnia, a genetic disorder leading to progressive insomnia and neurodegeneration (Lugaresi et al. 1986; Goldfarb et al. 1992).

Zebrafish have 2 prion gene orthologs, *prp1* and *prp2* (Cotto et al. 2005) (Table 1). Despite limited amino acid similarity to their mammalian counterparts, they still share significant 3D structural resemblance and expression patterns (Rivera-Milla et al. 2006). The presence of 2 orthologs with distinct and mostly non-overlapping expression patterns allows targeted exploration of the functions of PrP1 and PrP2 in different cell types (Cotto et al. 2005).

Zebrafish larvae lacking *prp1* have only mild phenotypes, similar to mammalian *Prnp* knockouts (Leighton et al. 2018a), but those lacking *prp2* exhibit increased seizure-like behavior following exposure to the convulsant pentylenetetrazol (Fleisch et al. 2013). Patch-clamp recordings to record miniature excitatory postsynaptic currents from intact hindbrains revealed that *prp2* plays a role in closing N-methyl-D-aspartate receptors, contributing to neuroprotection during glutamate-induced excitotoxicity (Fleisch et al. 2013). Unexpectedly, *prp1* loss reduced the seizure susceptibility seen in *prp2* mutants, and *prp1^−/−^; prp2^−/−^* double mutants have mild phenotypes, indicating opposing functions of these 2 orthologs rather than redundancy (Leighton et al. 2018a). *prp2^−/−^* mutants also have age-dependent memory decline, with 3-yr-old mutants performing poorly in an object recognition task, suggesting impaired cognitive appraisal (Leighton et al. 2018b). These findings highlight a conserved role for PrP in memory and establish zebrafish as a model to study PrP-related mechanisms in AD and prion diseases. However, gain-of-function zebrafish models of human PrP disease have to date not been attempted, likely due to the significant challenges in safe handling of such Prion species, which limits the degree to which disease forms can be studied in fish.

### Spinocerebellar ataxia

Spinocerebellar ataxias (SCAs) are a diverse group of autosomal dominant neurodegenerative disorders characterized by progressive gait ataxia, speech difficulties, limb incoordination, and oculomotor disturbances, largely due to cerebellar and brainstem degeneration (Sarasamma et al. 2023). More than forty causal genes have been identified, including CAG repeat-expansion genes and mutations in ion-channel and transcriptional regulators (Bhandari et al. 2023). Several SCAs have been modeled in zebrafish.

#### Polyglutamine (polyQ) SCAs

Spinocerebellar ataxia type 1 is caused by an expanded CAG repeat in Ataxin-1 (ATXN1). The 2 zebrafish ATXN1 homologs, *atxn1a* and *atxn1b*, are expressed in the developing cerebellum (Carlson et al. 2009). In zebrafish, overexpression of human ATXN1 with an expanded polyQ tract induces Purkinje cell abnormalities and locomotor deficits (Elsaey et al. 2021). Spinocerebellar ataxia type 3 (SCA3/Machado–Joseph disease) is caused by >40 CAG repeat expansions in ATXN3, which encodes a deubiquitinating enzyme involved in regulating protein stability and degradation (Riess et al. 2008). Injection of mRNA encoding 80 polyQ repeats of Atxn-3 into zebrafish embryos triggers apoptosis, predominantly in the CNS during early development (Liu et al. 2016). Similarly, Atxn3-84Q zebrafish exhibit reduced survival, *ataxin-3* neuropathology, and motor impairment (Watchon et al. 2017).

#### Ion-channel SCAs

Channelopathies represent a major SCA class. Mutations in *KCNC3*, which encodes the voltage-gated potassium channel Kv3.3, is responsible for Spinocerebellar ataxia type 13 (Waters et al. 2006). This channelopathy has been modeled in zebrafish by mRNA injections of a human disease-related dominant-negative form of KCNC3 (R420H) (Issa et al. 2011), which led to the suppression of excitability in fast-spiking motor neurons to cause impaired motor-coordination. Spinocerebellar ataxia type 6 (SCA6) is caused by mutations leading to >20 CAG repeats in the *CACNA1A* gene, which encodes the α1A voltage-dependent calcium channel subunit (Zhuchenko et al. 1997). A zebrafish *cacna1Ab* missense mutant called *fakir* lack touch sensation (Low et al. 2012), showing that perturbation of potassium and calcium channel function related to SCAs, even if not directly modeling the disease, is sufficient to generate ataxia-relevant phenotypes in vivo.

### Environmental causes of neurodegeneration

Environmental factors are increasingly recognized as contributors to the onset and progression of neurodegenerative diseases. Given the relative ease with which both larvae and adult zebrafish can be exposed to small molecule perturbagens, numerous neurotoxins associated with neurodegenerative disease, including pesticides and metals, have been explored for their neurodegenerative impact (recently reviewed in Yin and Horzmann 2024).

#### Pesticides/insecticides/fungicides

Exposure to the pesticide rotenone is known to cause loss of dopaminergic neurons leading to Parkinson's-like symptoms in humans (Betarbet et al. 2000). Similarly, rotenone exposure in both larval (Ranasinghe et al. 2024) and adult (Grisotto et al. 2025) zebrafish produce PD-related pathologies including neuronal loss and deficits in locomotion. Another herbicide, paraquat, also has been implicated as a PD risk factor (Tanner et al. 2011). In zebrafish, paraquat triggers the generation of reactive oxygen species (Liu et al. 2018) and affects mitochondrial bioenergetics, dopamine expression, and locomotor activity (Wang et al. 2018), phenotypes that may be relevant to PD.

#### Heavy metals

A wide variety of heavy metal toxicants, including lead, arsenic, cadmium, mercury, manganese, and aluminum, have been implicated in neurodegenerative disease, with varying degrees of evidence for causation (Bakulski et al. 2020; P. Chen et al. 2016). As in rodents, zebrafish exposed to a variety of metals have impaired motor behaviors, cerebellar abnormalities, and increases in neuronal oxidative stress. For example, combination exposure of larval fish to lead and arsenic can alter swim speed (Liu et al. 2024), acute manganese exposure reduces activity and causes loss of postural control (Alba-González et al. 2024), and sub-lethal exposure to mercury results in a subtle hyperlocomotion phenotype (Henriques et al. 2023). More work testing the impact of metal exposures in genetic disease models (gene–environment interactions) would help craft a clearer picture of how these toxins impact the initiation and progression of neurodegeneration.

## Zebrafish phenotyping pipeline for systematic analysis of neurodegenerative risk genes

So far, we have seen several examples of zebrafish genetic manipulations of neurodegenerative disease pathways. However, with few exceptions, these studies have mostly focused on well characterized disease genes, usually tackling just one gene at a time. These experiments have also been relatively narrow in scope, with a major goal being to show functional conservation of disease proteins in zebrafish and to make some initial phenotypic characterizations in early embryos. However, the true power of the zebrafish over other vertebrate systems lies in taking full advantage of the available medium and high-throughput methods to manipulate many genes in parallel and in combination while systematically assessing phenotypes and screening for small molecule mediators. Such approaches have already been applied to neurodevelopmental disorders like schizophrenia (Thyme et al. 2019) and autism (Mendes et al. 2023) and more recently have been used for genes involved in early and late onset AD (Kroll et al. 2025).

**Fig. 1.**
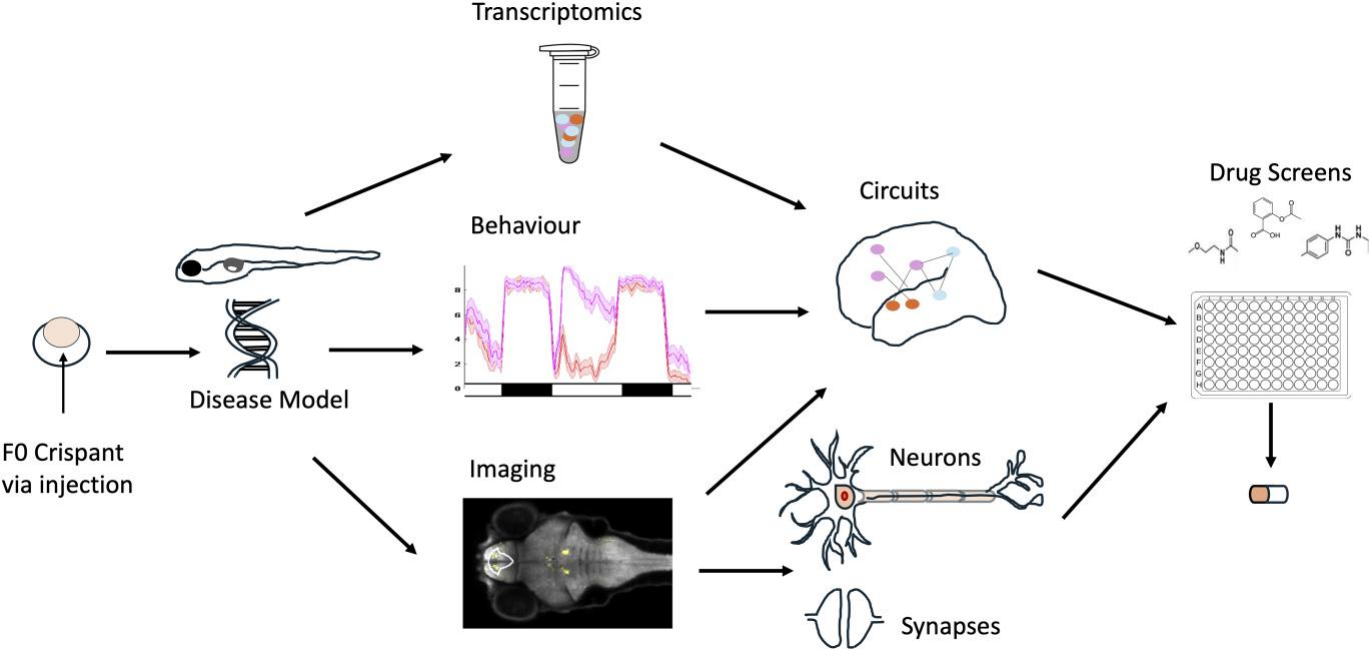
Zebrafish pipeline for dissecting cells and molecules underlying disease mechanisms. Zebrafish mutants can be quickly generated and run through phenotyping pipelines to discover druggable targets.

In the following sections, we outline a complete pipeline (Fig. 1) to map zebrafish risk genes to phenotypes and druggable pathways, highlighting key experimental and theoretical considerations at each of the major steps of the pipeline.

### IDing causal neurodegenerative disease-risk gene orthologs

The availability of well-annotated zebrafish and human genomes, along with tools such as the ENSEMBL database and reciprocal BLAST, makes identifying zebrafish orthologs of human genes straightforward. The Zebrafish Genome Project provided a high-quality sequence assembly of the approximately 1.4 Gb haploid zebrafish genome, organized as 25 chromosomes (Daga et al. 1996; Howe et al. 2013). Other online resources (Table 2), including the Zebrafish Information Network, Zebrafish International Resource Center (ZIRC), and European Zebrafish Resource Center (EZRC), provide researchers with comprehensive genomic and phenotypic data to facilitate the identification and functional analysis of zebrafish orthologs of human disease genes.

**Table 2.** Major zebrafish experimental resources.

General resources	Description	Website	Reference
Zebrafish Information Hub	A central database for zebrafish research resources	https://zfin.org	Sprague et al. (2001)
Zebrafish International Resource Center (ZIRC)	Provides zebrafish lines and husbandry resources	https://zebrafish.org/home/guide.php	
European Zebrafish Resource Facility (EZRC)	Provides zebrafish lines, genetic tools, and advanced phenotyping resources	https://ezrc.kit.edu	Geisler et al. (2016)
National Zebrafish BioResource Project	Japanese zebrafish repository offering resources for genetic and developmental research	http://www.shigen.nig.ac.jp/zebra/index_en.html	Okamoto and Ishioka (2010)
Zebrafish Disease Models Society	Promotes the use of zebrafish as models for human diseases	https://www.zdmsociety.org	
Zebrafish Insertion Collection (ZInC)	Database of retroviral insertional mutants	http://research.nhgri.nih.gov/zinc	Varshney et al. (2013)
Genomic resources			
Zebrafish Genome Resource (Ensembl)	Zebrafish genome, including annotated genes and comparative genomics tools	https://www.ensembl.org/Danio_rerio/Info/Index	Howe et al. (2013)
Zebrafish Genome Database (NCBI)	A comprehensive genome database maintained by the National Center for Biotechnology Information (NCBI)	https://ncbi.nlm.nih.gov/genome?term=danio%20rerio	Sayers et al. (2021)
UCSC Genome Browser	A comprehensive genome broswer	https://genome.ucsc.edu/	
DANIO-CODE	Database of annotated zebrafish functional genomic elements	http://danio-code.zfin.org	Baranasic et al. (2022)
Brain atlases			
Mapzebrain	A reference brain atlas with standardized brain region annotations, markers & transgenes	https://mapzebrain.org/home	Kunst et al. (2019)
Zebrafish Atlas	A reference larval zebrafish brain atlas for functional and anatomical studies	https://zebrafishexplorer.zib.de/home/	Randlett et al. (2015)
UCL Zebrafish Brain	Anatomical description of larval zebrafish brain regions	https://zebrafishucl.org/zebrafishbrain	Turner et al. (2014)
Adult Zebrafish Brain Atlas (AZBA)	A reference adult zebrafish brain atlas	http://www.azba.wayne.edu	Kenney et al. (2021)
Other resources			
ZOLTAR	Online tool to predict druggable pathways from zebrafish behavioral fingerprints	https://francoiskroll.shinyapps.io/zoltar/	Kroll et al. (2025)
zTrap	Database of gene traps and enhancer traps	http://kawakami.lab.nig.ac.jp/ztrap/	Kawakami et al. (2010)
Zebrahub	Single-cell RNA sequencing and live light-sheet imaging of zebrafish development	https://zebrahub.sf.czbiohub.org/	Lange et al. (2024)
ZCL & ZCDL	Single-cell RNA sequencing data for major zebrafish organs	https://bis.zju.edu.cn/ZCL/	Jiang et al. (2021), Wang et al. (2023)
Daniocell	Single-cell gene expression data generated from whole-animal zebrafish embryos and larvae across 62 stages of development	https://daniocell.nichd.nih.gov/	Sur et al. (2023)

One critical issue is to prioritize studying the correct causal disease risk genes, especially those uncovered by GWAS studies. This is challenging because each genetic risk locus often contains dozens of candidate genes that may be the relevant causal gene; moreover, the complex regulatory architecture of the genome means that noncoding variants can affect genes quite far from their physical location. Several strategies have been developed to address this problem, including expression quantitative trait locus mapping, fine-mapping of regulatory single-nucleotide polymorphisms, and integrative multi-omics approaches (Zhang et al. 2024). Methods that probe the local genome architecture in disease-relevant cell types, including ATAC-seq to map accessible chromatin domains and Capture C to assess local 3-D genomic interactions, can be particularly successful in pinpointing likely causal genes within a locus. One recent example used these tools to dissect a GWAS locus associated with insomnia, pinpointed phosphatidylinositol glycan (PIG)-Q as the likely causal gene, and then validated insomnia phenotypes in both Drosophila and zebrafish (Palermo et al. 2023). However, none of these approaches are fool-proof, and ambiguity as to which is the risk-causative gene persists for several neurodegenerative disease loci. For example, in PD, the 4p16.3 locus (the TMEM175/GAK/DGKQ/IDUA block) is one of the top risk loci, yet different multi-omic fine-mapping efforts have nominated TMEM175, GAK, or IDUA as the disease-causing gene, leaving this region categorized as a multi-gene locus (Yang et al. 2020).

Given these uncertainties in assigning causal genes within complex loci, the scalability of zebrafish genetics offers an alternative approach to directly test each candidate gene within a locus. Techniques such as CRISPR F0 mutagenesis (see the *loss of function* section below) are robust and scalable to generate loss-of-function mutations for multiple candidate genes within a locus, alone or in combination, without presupposing which one is responsible. Such strategies have already been successfully used in zebrafish to dissect complex neurodevelopmental loci, such as the human 16p11.2 interval (Qiu et al. 2019), and could similarly be applied to neurodegenerative disease loci. Such disease loci knockout screens could be performed on transgenic backgrounds overexpressing toxic aggregating proteins to enable the search for genetic modifiers. Such an approach has already been successful in screening for novel genetic modifiers of *HTT* CAG somatic expansion in a mouse model of HD (Genetic Modifiers of Huntington's Disease (GeM-HD) Consortium 2025), and zebrafish would be an ideal model in which to perform similar screens at scale.

To support prioritization of candidate genes, examining the expression patterns of identified zebrafish orthologs can be informative. Spatial and cell-type–specific expression can be assessed using high-throughput methods such as hybridization chain reaction (HCR) (Dirks and Pierce 2004), which enables multiplexed in situ detection across brain regions. Publicly available single-cell RNA-sequencing datasets (Table 2) also allow investigation of ortholog expression at cellular resolution across developmental stages (Jiang et al. 2021; Lange et al. 2024). Together, these tools provide complementary, high-resolution information to guide gene selection and interpretation in the context of neurodegeneration-relevant tissues.

### Generating loss- and gain-of-function mutants in candidate disease genes

Once candidate disease gene orthologs have been identified, a variety of methods can be employed to eliminate (loss of function) or modify gene activity through overexpression or misexpression of disease-related mutations (gain of function). Here, we discuss the major methods employed for generating both classes of zebrafish mutants for the analysis of neurodegenerative disease, including historical and modern methodologies.

#### Loss of function

Prior to the development of modern gene editing tools, a major way to knock down gene function was using morpholinos (MOs), which are chemically modified antisense oligonucleotides that block translation or splicing of specific mRNAs. While often effective for probing early developmental roles, the temporary nature of MO-induced knockdown (since the MO degrades and dilutes over developmental time) made it less suitable for studying phenotypes past 5 d post fertilization (dpf) (Veldman and Lin 2008), when many important zebrafish behaviors emerge. Moreover, concerns over off-target and non-specific effects have significantly limited their current use (Robu et al. 2007). In particular, although many, often quite severe, developmental phenotypes have been reported in MO-based knockdown studies of neurodegeneration-related genes, such as *appa, appb* (Joshi et al. 2009), and *lrrk2* (Sheng et al. 2010), these have often not been replicated in studies using genetic mutants (Banote et al. 2020; Ozcan et al. 2024; Kroll et al. 2025) (see also the section, *Neurodegenerative disease genes and zebrafish models*).

Many gene-editing technologies have been employed in zebrafish, including zinc finger nucleases, transcription activator-like effector nucleases, and even brute force chemical mutagenesis and sequencing approaches such as targeting-induced local lesions in genomes (TILLING). Thousands of mutant lines generated with these methods are available directly through resources such as the Sanger Institute, ZIRC or EZRC (Table 2). However, the simple design, few required components, and scalability due to high in vivo cutting efficiency of the CRISPR/Cas9 system have made it the system of choice for targeted editing. Composed of a DNA nuclease (Cas9) and a guide RNA (a stem-loop tracrRNA and programmable crRNA) that directs Cas9 activity to specific genomic sequence targets, the CRISPR-Cas9 system is easily injected into the yolk at the 1-cell stage to generate double strand breaks that lead to frameshift mutations at the targeted site (Hwang et al. 2013).The efficiency of cutting and frameshift generation is very high; even the original zebrafish CRISPR-Cas9 demonstration achieved cutting efficiencies upwards of 50% (Hwang et al. 2013), allowing for the generation of novel mutant lines with relatively few animals needed for screening (Varshney et al. 2015; Burger et al. 2016). Given these advantages, mutants in a variety of neurodegenerative disease genes, including *app*a/*appb*, *clusterin* (*clu)*, *sortilin related receptor 1* (*sorl1*), and the *prion protein* (*prp1/2)* have already been generated using CRISPR/Cas9 gene editing (Fleisch et al. 2013; Ozcan et al. 2020; Kroll et al. 2025).

The high efficiency and multiplexing capabilities of CRISPR-Cas9 have opened up unprecedented opportunities for rapid and scalable knockout experiments in zebrafish. For example, multiple guide RNAs that target distinct genes can be co-injected into the same egg to generate founder lines that harbor loss-of-function mutations in several genes simultaneously. One study generated zebrafish families with mutations in up to 5 schizophrenia-associated genes each, enabling highly efficient testing of the effects of loss of function in zebrafish orthologs of 132 human schizophrenia-associated genes across a host of behaviors (Thyme et al. 2019). Other studies have used Crispr multiplexing to quickly generate mutants in classes of genetic targets, including 32 DNA repair and replication genes (Shin et al. 2021), 19 Franconi anemia risk genes (Ramanagoudr-Bhojappa et al. 2018), and 5 genes related to hearing loss (Guo et al. 2022).

Pushing the efficiency even further, recent work has shown that co-injection of Cas9 protein incubated with 3, or even as few as 2, guide RNAs that target the same gene at different exons can lead to fully penetrant, bi-allelic loss-of-function knockouts in the injected animal (Shah et al. 2015; Kroll et al. 2021; Quick et al. 2021). Since many of the F0 knockout or “crispant” animals tested thus far recapitulate phenotypes observed in traditional stable loss-of-function mutants (Wu et al. 2018; Watson et al. 2020; Quick et al. 2021; Benoit et al. 2024; Kroll et al. 2025), this approach offers advantages of speed and experimental flexibility. For example, crispants can be made directly in different transgenic or mutant backgrounds, thereby avoiding the (often) complex breeding schemes necessary to pair genetic mutants with fish containing multiple transgenic reporters (Kroll et al. 2021). Similarly, crispants can be generated in parallel on multiple strains to test whether mutant phenotypes are influenced by different genetic backgrounds (Bretaud et al. 2011; White et al. 2022). Crispr F0 approaches also afford a rapid way to examine for functional redundancy in duplicated genes by simultaneous targeting (Kroll et al. 2025). Recent technological advances such as MIC-Drop, in which DNA barcodes and multiplexed CRISPR ribonucleoproteins are assembled in nano-droplets, allow for even greater throughput for functional genetic screens (Parvez et al. 2021, 2023). Using MIC-Drop, Crispr F0 mutants in 188 genes were quickly tested for novel cardiac phenotypes, with the potential to increase the screening to thousands of genes (Parvez et al. 2021).

These zebrafish CRISPR F0 knockout screens offer a rapid approach to probe the functions of neurodegenerative disease risk genes. One recent screen targeted a panel of early- and late-onset AD risk genes and revealed that reduced nighttime sleep was a common phenotype in larval zebrafish (Kroll et al. 2025). This aligns with mounting evidence from human studies suggesting that sleep disruption may not only be a symptom but also a contributing factor to AD pathogenesis, even in early life stages (Shokri-Kojori et al. 2018; Holth et al. 2019; Wong and Lovier 2023; Jin et al. 2025). Zebrafish CRISPR F0 screening therefore offers a scalable platform to uncover early behavioral signatures of disease risk that mechanistically precede the clinical onset of symptoms.

The flexibility of the CRISPR-Cas9 system allows for more sophisticated spatially and temporally restricted manipulations than simply generating whole animal knockouts. Pairing a transgenic U6 promoter (a strong RNA polymerase III promoter that ensures efficient expression of small RNAs) to drive gene-targeting guide RNAs with transgenes that restrict Cas9 expression to select tissues or cell types can inactivate target genes just in those tissues (Ablain et al. 2015). Similarly, the Cre-Controlled CRISPR (3C) system incorporates a zebrafish U6a promoter that constitutively drives the gRNA while regulating Cas9-GFP expression with Cre, which enables targeted, inducible gene inactivation in specific tissues with fluorescent labeling of mutated cells (Hans et al. 2021). Other transgenes, such as heat shock or drug-inducible promoters that drive *cas9* expression, give experimental control over the timing of Crispr mutagenesis, which is useful to avoid disruption of early developmental events (Li et al. 2016). Although these spatial and temporal tools have not yet been extensively used to investigate neurodegenerative disease in the zebrafish model, several interesting applications come to mind. Zebrafish have reliable transgenic reporter lines that restrict expression to neurons or glia, which could be used to interrogate cell-type specific roles of neurodegenerative disease risk genes. Additionally, selective activation of CRISPR-based gene editing in older brain tissues could be used to model the effects of disease-related somatic mutations, the accumulation of which has been hypothesized to contribute to disease progression in some contexts (Lodato et al. 2018).

Other CRISPR variants, such as CRISPR-Cas13, selectively target mRNA instead of DNA and may therefore have enhanced therapeutic potential because they avoid more permanent genetic changes (Kushawah et al. 2020). A recent example of this approach in zebrafish is the study by Kempthorne et al. (2025), which demonstrated the therapeutic potential of CRISPR-Cas13d (CasRx) in both zebrafish and rodent models of ALS. In the zebrafish, overexpression of toxic C9orf72 repeat RNAs causes behavioral hyperactivity, a phenotype that was robustly rescued by co-expression of CasRx and C9orf72-targeting guide RNAs (Kempthorne et al. 2025). This provides in vivo proof of concept for using CRISPR-based RNA targeting to counteract toxic RNA species in C9orf72-linked ALS/FTD.

#### Gain of function

Unlike loss-of-function mutations, which can be performed in a massively parallel manner, gain-of-function manipulations in zebrafish require bespoke alterations that introduce modified neurodegenerative disease genes with point mutations or truncations that affect protein function and/or aggregation. There are 2 major methods for introducing gain-of-function mutations in zebrafish: transgenic overexpression of genes encoding mutated, often humanized, proteins and precise gene editing to directly change the sequence of the endogenous zebrafish gene into disease-associated human forms. Both of these methods have practical uses.

The generation of zebrafish over-expression transgenic lines requires only a simple injection of plasmids that contain promoter elements driving gene-of-interest expression, usually paired with fluorescent reporters. The constructs are mobilized into the genome by transposon-based systems such as Tol2, which leads to random integration at multiple genomic loci (Kawakami 2005). While this greatly boosts the transgenesis success rate, random integration of multiple copies can lead to variable expression due to position effects and transgene silencing (Ni et al. 2008). Even when using the same transgene construct in the same organism, studies in neurodegeneration research have reported divergent phenotypes due to these subtle, yet significant, biological variables. For instance, in the widely used SOD1-G93A ALS model, high-copy and low-copy lines exhibit drastically different disease onset and severity, even though they carry the same mutated SOD1 sequence (Acevedo-Arozena et al. 2011).

To overcome these limitations, the recently developed pIGLET system (Lalonde et al. 2024) instead uses phiC31 integrase for targeted transgenesis into attP landing sites (pIGLET14a and pIGLET24b). These attP landing sites are engineered into predefined genetic loci chosen to avoid heterochromatin or nearby enhancers that can alter transgene expression, thereby ensuring consistent expression across multiple transgenic lines, even in the injected F0 embryos. Thus, the pIGLET system is well-suited to ensure consistent expression levels of normal and toxic proteins needed for applications such as systematic testing of copy number variants associated with disease (Moyer and Thyme 2025), like the extra copy of APP that arises from trisomy 21 (Down's syndrome) and leads to accelerated amyloid plaque formation and dementia in patients (Head et al. 2015).

A fairly large set of flexible transgenic tools are available in zebrafish to enable precise temporal and spatial mis-expression of genes. Combinatorial genetic methods such as the Gal4/UAS, Cre/lox, and the Q systems allow for rapid pairing via simple breeding of tissue- or temporal-specific driver lines with mis-expression and reporter lines to precisely control gene expression. The separate propagation of driver and effector lines permits studies of otherwise lethal phenotypes by controlling the timing of gene activation, a particularly useful feature for studying toxic gain-of-function neurodegenerative disease products. One study used a pan-neuronal Gal4 driver line to mis-express a human pathogenic variant of Tau (hTAU-P301L) under UAS control (Paquet et al. 2009). The expression of hTAU-P301 in neurons had several AD-like phenotypes, including delayed neuronal differentiation and behavioral abnormalities such as severely altered escape response to a touch stimulus. In a second example, the lethality of overexpressing Htt with polyQ expansion in transgenic zebrafish was bypassed by creating Cre-dependent Htt-25Q and Htt-97Q transgenes (Veldman et al. 2015) that could be temporally activated at later developmental stages by a heat shock-inducible Cre recombinase. These Htt-overexpressing fish developed Htt protein aggregates but survived to adulthood and developed progressive motor impairments only after 16 wk of age.

Direct gene editing of endogenous neurodegenerative disease genes to make toxic versions that mimic human mutations or to tag proteins for trafficking studies is also now possible in zebrafish. Numerous methods use CRISPR-Cas9 to create targeted double strand breaks that stimulate homologous recombination between the endogenous genetic locus and exogenously supplied DNA constructs containing desired point mutations, tags, or other modifications to generate “knock-in” alleles (Kimura et al. 2014). Some of these knock-in methods, such as GeneWeld (Welker et al. 2021) or cloning-free, PCR-based methods (Mi and Andersson 2023) achieve high enough efficiency to easily screen for germline inheritance of the knock-in alterations. Other knock-in methods developed for zebrafish include DNA base editors and CRISPR Prime editors, both of which make targeted genetic alterations without cutting DNA, reducing off-target effects. DNA base editors come in 2 main flavors, in which a non-cleaving Cas9 is fused to either a cytidine or adenine deaminase to generate guideRNA-directed conversions of single base pairs (eg C→T or G→A) in target sequences (Komor et al. 2016; Zhang et al. 2017). CRISPR prime, which uses a Cas9-nickase fused to a reverse transcriptase to direct local sequencing edits, is capable of making even more complex, precise edits than single base conversions and is reported to generate somatic mutations at rates up to 30% in zebrafish (Petri et al. 2022).

Although these various knock-in strategies have not, to our knowledge, been used to model neurodegenerative diseases in zebrafish, these applications have already had great success in other animal and human cell-based models. For example, a CRISPR-generated knock-in of a human disease mutation into the endogenous mouse *Tardbp* gene identified TDP-43 gain of function as a pathogenic mechanism that may drive ALS-FTD (White et al. 2018). Similarly, unlike traditional overexpression models, homozygous MAPT P301L knock-in mice did not develop overt tau pathology during their lifespan; instead, they showed reduced tau phosphorylation and subtle, age-dependent changes such as increased mitochondrial transport and spontaneous activity that may be relevant early physiological consequences of tau dysfunction in FTD (Gilley et al. 2012). Similar knock-in approaches in zebrafish would be a potent way to make new neurodegenerative disease models for brain and behavioral phenotyping.

### Mutant phenotyping

Although complex genetic manipulations are now straightforward in many species, significant bottlenecks still exist when trying to map genes to phenotypes and functions. These efforts are greatly facilitated in zebrafish by tools that enable cell-type specific observations of global transcriptomic changes, quantitative phenotyping of subtle behaviors, and live imaging and manipulation of neurons to experimentally probe circuits governing behavior. Since most of these tools focus on phenotyping at early larval stages, one may wonder if these methods are appropriate for modeling late-onset neurodegenerative diseases. However, an important but often neglected concept is that superficially unrelated phenotypes can nevertheless pinpoint key underlying disease mechanisms. For example, unbiased phenotyping of ALS animal models revealed that immune dysfunction and inflammation arise at an early stage even before motor symptoms appear (Atanasio et al. 2016; O’rourke et al. 2016), highlighting an unexpected microglial contribution to disease progression.

In the following sections, we describe major single cell transcriptomics, behavioral, and imaging approaches in zebrafish, with a particular emphasis on how these can be used to map the functions of neurodegenerative disease genes onto specific cellular and signaling pathways that regulate the brain.

#### Bulk and single cell transcriptomics/genomics

Bulk and single-cell transcriptomics have rapidly become integral to zebrafish phenotyping pipelines. When combined with genetic and behavioral data, these approaches can link candidate neurodegeneration risk genes to disrupted cell states or neural circuits. For example, transcriptome analysis of zebrafish carrying an early-onset familial AD-like mutation (*psen1Q96_K97del*), previously shown to affect mitochondrial function and endolysosomal acidification in adult brains, revealed disruptions in oxidative phosphorylation, extracellular matrix functions, and iron homeostasis as early as 7 dpf (Dong et al. 2021). Well established single-cell transcriptomic methods supported by robust analysis pipelines such as Seurat (Butler et al. 2018) or Scanpy (Wolf et al. 2018) extend the resolution to the cellular level, enabling profiling across tissues in a variety of developmental stages and disease contexts. In one example, single-cell and single-nucleus transcriptomic comparison between a zebrafish amyloid toxicity model and human brain samples uncovered both conserved neuronal responses and zebrafish-specific regenerative astroglial transcriptional signatures related to amyloid exposure (Cosacak et al. 2022). Complementary assays such as scATAC-seq provide even more information on chromatin accessibility and epigenomic dysregulation, and have been applied successfully in zebrafish (Tornini et al. 2023), although not yet in neurodegenerative disease models.

#### Behavioral phenotyping

**Fig. 2.**
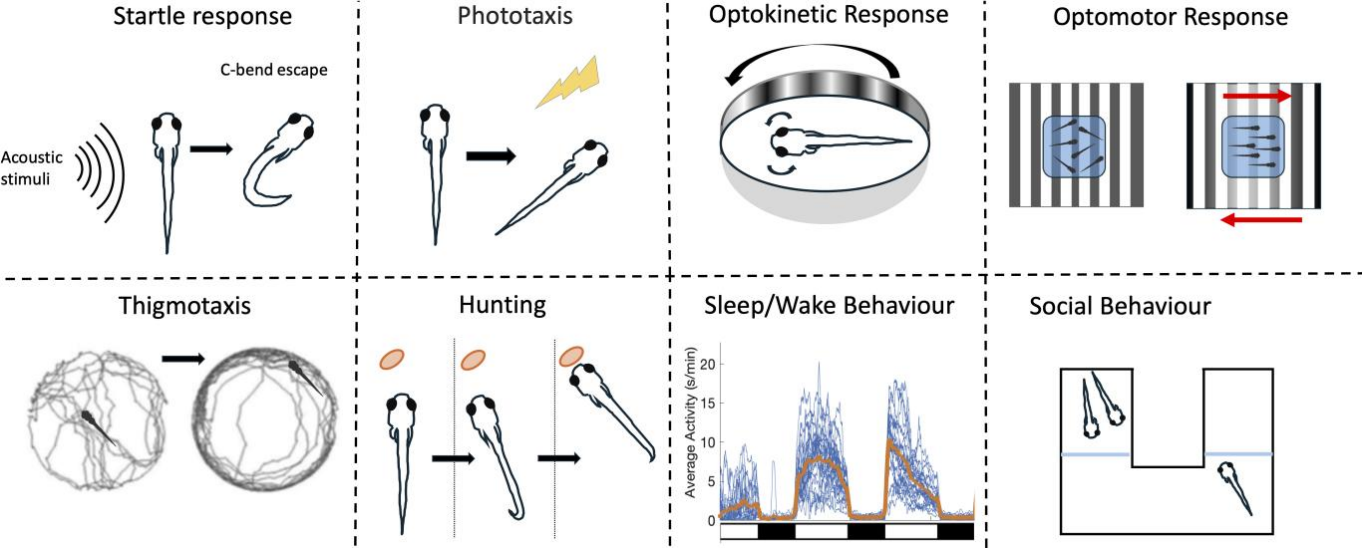
Common behavior assays used in zebrafish. A variety of simple and complex behavioral assays can be used to map circuit dysfunction in neurodegenerative disease models.

Quantitative phenotyping of larval behavior can uncover even subtle functional consequences of genetic alterations linked to neurodegenerative diseases. Standard zebrafish assays range from simple, stimulus-evoked responses to complex, longer-term behaviors (Fig. 2). Many of these behaviors, such as motor coordination and sleep, have a clear direct relevance to neurodegenerative disease, while others have a more indirect relationship. However, since these behaviors rely on distinct sets of neuronal circuits and pharmacologically tractable signaling cascades, assessing multiple behavioral outputs can reveal disruption in important neuromodulatory systems, including the serotonergic, dopaminergic, or noradrenergic populations commonly affected in neurodegenerative disease. Sequential testing of different assays in the same animals (Kokel et al. 2010; Thyme et al. 2019) further enhances screening throughput and the power of computational analyses to find mechanistic links that map across behaviors.

Recent computational advances have transformed the scale and granularity of behavioral phenotyping. For example, Ghosh and Rihel (2020) demonstrated that an unsupervised learning pipeline can analyse long-timescale larval zebrafish tracking data to organize millions of individual locomotor bouts into distinct behavioral motifs. This framework captures structure across timescales from milliseconds to 24-h circadian rhythms and sensitively detects changes caused by genetic mutations or pharmacological interventions. Complementing this approach, deep learning-based tools such as DeepLabCut can track body posture during individual swims at a high resolution (Mathis et al. 2018). This type of kinematic phenotyping, in which tail-beat amplitudes, frequency, and curvature dynamics are extracted and clustered into discrete swim motifs, has been used in several studies that show how subtle alterations in motor control can be detected even when overall locomotor levels appear normal (Severi et al. 2014; Banerjee et al. 2022; Mackensen et al. 2025; Scholz et al. 2025).

Other approaches take full advantage of the ability to quickly assay multi-dimensional phenotypes to generate mutant and drug-induced behavioral fingerprints across a range of behavioral assays. Early approaches organized sleep–wake and light-driven behavioral parameters that were quantifiably altered by thousands of small molecules into behavioral fingerprints. These fingerprints could then be clustered for rapid classification and prediction of molecular targets (Kokel et al. 2010; Rihel et al. 2010b), including the identification of novel antipsychotic compounds (Bruni et al. 2016), based solely on behavioral similarity. Similar fingerprinting approaches have been used to identify molecular targets of environmental toxins (Owen et al. 2025) and altered sensitivities to drugs in genetic mutants of AD risk genes (Kroll et al. 2025) (See also, *Small molecule screening and Behavioral clustering*).

Although not all of the major zebrafish behaviors have been analyzed in models of neurodegenerative disease, their ability to be sequentially interrogated to capture the function of diverse neuronal classes makes them highly relevant for future work. Integrating pose-level kinematics, unsupervised behavioral segmentation, and multidimensional fingerprints into an analytic pipeline will enable the construction of neurodegeneration-related phenotype atlases that capture circuit-level vulnerabilities across timescales. Here, we summarize major larval behavioral phenotypes and highlight aspects relevant to neurodegenerative disease.

### Stimulus-evoked responses—well defined circuits and pharmacology

#### Escape swimming

Unfolding over just milliseconds, the startle response is among the fastest behaviors characterized in vertebrates. This behavior is triggered by tactile or acoustic stimuli delivered to the head or tail and is mediated by a core circuit centered on the large Mauthner neuron in the hindbrain (Kimmel et al. 1974; Eaton and Farley 1975), with additional circuits recruited for forceful responses to head-directed stimuli (O’malley et al. 1996; Liu and Fetcho 1999). This core circuitry is modulated by multimodal upstream inputs, including the visual system and the lateral line, which is involved in sensing water flow (Mchenry et al. 2009; Temizer et al. 2015; Dunn et al. 2016). These escape circuits use both excitatory (glutamatergic) and inhibitory (glycinergic) transmission to direct stereotypical startle patterns (Ganser and Dallman 2009) and 3-D swim trajectories (Stewart et al. 2015). The startle response also exhibits robust NDMA-receptor-dependent habituation to repeated stimuli (Marsden and Granato 2015). These features make the startle circuit a direct and sensitive readout of sensorimotor integration, circuit resilience, and synaptic plasticity– all processes that are disrupted by neurodegenerative disease.

#### Optomotor and optokinetic response

The optomotor response (OMR) is an innate behavior in which zebrafish swim in the direction of optical flow, such as that generated by unidirectionally moving stripes (Fleisch and Neuhauss 2006). The OMR depends on complex sensorimotor integration in retinal, tectal, and hindbrain motor output circuits (Fleisch and Neuhauss 2006; Severi et al. 2014; Naumann et al. 2016), making this behavior useful for probing sensory-motor coordination. The OMR's sensitivity to dopaminergic modulation (Jha and Thirumalai 2020) makes it a good assay for evaluating dopamine integrity relevant to neurodegenerative disease such as Parkinson's. The optokinetic response (OKR), in which larvae track moving scenes with smooth eye pursuits, provides another clean readout of visuomotor integration, which can be disrupted by neurodegenerative disease processes. For example, the altered OKR responses that were found in a zebrafish tauopathy model recapitulated eye-movement defects seen in neurodegenerative conditions like Progressive Supranuclear Palsy (Bai et al. 2024).

#### Responses to light-dark transitions

The visual-motor response (VMR) is a rapid and sustained increase in swim speed and duration following a sudden transition from light to dark. This behavior was initially attributed to visual input but was later shown to depend strongly on non-retinal, extraocular photoreception (Emran et al. 2008; Fernandes et al. 2012). The simplicity, quantifiability, and high throughput of this assay have made it a staple in toxicology, pharmacology, and genetic studies of arousal and sensorimotor function (Kokel et al. 2010; Rihel et al. 2010b). The VMR has also been used to detect subtle neural deficits in models of neurodegeneration and then to search for interventions that could reverse these degeneration phenotypes. For example, a zebrafish *wfs1* knockout model (*wfs1ab^KO^*) of Wolfram Syndrome, a rare neurodegenerative disease, had hypersensitivity to light–dark transitions that could be restored by *NCS1* mRNA injections into the eggs (Crouzier et al. 2022). In another example, reduced light-startle and VMR responses in a model of retinal neurodegeneration were rescued by small molecules, thereby suggesting carvedilol as a potential treatment for retinitis pigmentosa (Ganzen et al. 2021).

### Complex behaviors—higher order brain functions

#### Sleep and circadian rhythms

Continuous multi-day, high-throughput tracking of spontaneous swimming reveals robust diurnal sleep–wake cycles in zebrafish as young as 5 d post fertilization (Rihel et al. 2010b). Sleep in larvae is defined as a period of behavioral quiescence lasting at least 1 min, as larvae during such an inactive bout exhibit an increased arousal threshold to multiple types of stimuli (Zhdanova et al. 2001; Prober et al. 2006). Zebrafish sleep is homeostatically regulated such that periods of forced wakefulness are followed by increases in sleep. Larval sleep is also regulated by the 24-h circadian clock, with the most consolidated sleep occurring at night, even under constant conditions (Gandhi et al. 2015; Barlow and Rihel 2017). Larval sleep is also governed by neuronal circuits, including the wake-promoting hypocretin/orexin system, and molecular signaling molecules, such as sleep-promoting melatonin, that are known to modulate sleep in humans (Rihel et al. 2010a). Sleep in zebrafish has been shown to affect cognitive performance (Pflitsch et al. 2024), synapse formation and elimination (Suppermpool et al. 2024), DNA damage repair (Zada et al. 2019), and other processes that are important for healthy brain function. Moreover, many studies have linked early sleep disruptions to the progression and exacerbation of neurodegenerative diseases, including in AD (Sterniczuk et al. 2013; Holth et al. 2019), PD (Kashiwagi et al. 2024), and Creutzfeldt-Jakob disease (Wieser et al. 2006), and recent zebrafish work has shown that mutations in AD-risk genes or perturbation of Amyloid-β formation can similarly disrupt normal sleep structure even in young larvae (Ozcan et al. 2020, 2024; Kroll et al. 2025).

#### Thigmotaxis

Wall-hugging behavior (thigmotaxis) emerges as early as 5 dpf in zebrafish (Colwill and Creton 2011) and is associated with anxiety-like states that are modulated by serotonin, particularly through 5-HT1A receptors, as in other species (Herculano and Maximino 2014). Zebrafish thigmotaxis is sensitive to anxiolytic and anxiogenic interventions (Ahmad and Richardson 2013), making it a reliable index of anxiety. Altered thigmotaxis in zebrafish neurodegeneration models may therefore reflect dysfunctions in serotonin signaling or spatial circuit activity, 2 processes that are often altered in neurodegenerative diseases, including AD and PD. In one example, *lrrk2* KO fish showed normal thigmotaxis behavior, suggesting that *lrrk2* might not be important for non-motor behaviors associated with PD (Suzzi et al. 2021), an idea that awaits further study.

#### Hunting/prey capture

Prey capture is a complex behavior that requires sensory processing (prey detection), decision-making (orientation and approach), and precise motor execution (capture) (Muto and Kawakami 2013). These stereotyped hunting sequences can be readily tracked and quantified with high-speed cameras to probe for defects in sensory integration and motor planning. Because the underlying circuits are relatively well mapped, the assay offers a way to connect behavioral phenotypes to discrete neural mechanisms. For instance, cholinergic neurons in the optic tectum are engaged during prey detection (Corradi and Filosa 2021), while a small cluster of cholinergic neurons in the nucleus isthmi (NI) is critical for sustaining pursuit and capture (Henriques et al. 2019). Although prey capture has not yet been studied in zebrafish models of neurodegenerative disease, the combination of a quantifiable behavioral sequence and defined circuit components makes this assay an attractive approach to interrogate how neurodegeneration disrupts sensorimotor integration and sequential decision-making.

#### Social behavior

Social affiliation emerges early in zebrafish development, with shoaling behavior and preference for conspecifics detectable around 3 wk post-fertilization (Dreosti et al. 2015). Neuromodulators such as oxytocin and serotonin have been implicated in the development and expression of zebrafish social preferences, with disruptions in oxytocin and dopamine signaling altering social attraction (Gemmer et al. 2022). In particular, antagonism of D1 dopamine receptors reduces social preference in larval zebrafish (Scerbina et al. 2012), with similar results in mammals. Disruptions in D1 receptor signaling has been implicated in neurodegenerative disorders including AD and HD (Weeks et al. 1996; Kemppainen et al. 2000; Andre et al. 2011). Altered social engagement in zebrafish neurodegeneration models may therefore reflect deficits in motivation, sensory integration, or social cognition, which are all core features affected in various conditions, including frontotemporal dementia and PD.

### Brain imaging and manipulation

Larval zebrafish are ideal for in vivo brain imaging, allowing repeated, non-invasive, real-time observations of neuronal structure and function at single-cell resolution. Modern imaging modalities provide complementary strengths; for example, 2-photon microscopy enables deep tissue imaging with minimal photodamage, confocal microscopy offers optical sectioning for detailed cellular architecture, and light-sheet and multiphoton microscopy allow sub-second whole-brain volumetric imaging, even in freely behaving, untethered fish (Ahrens et al. 2013; Renninger and Orger 2013; Cong et al. 2017; Cho et al. 2023). Combined with genetically encoded sensors, these tools permit direct monitoring of neuronal calcium dynamics, neurotransmitter release, and other signaling events during behavior (Panier et al. 2013; Portugues et al. 2014; Muto and Kawakami 2016).

Advances in microscope automation further expand the experimental possibilities. For example, the vertebrate automated screening technology system not only streamlines larval handling and orientation for imaging but also enables large-scale, unbiased phenotyping by rapidly cycling hundreds of individuals through identical imaging pipelines (Chang et al. 2012; Pulak 2016). This capacity allows for genetic or pharmacological screens of circuit function, bridging high-throughput discovery with mechanistic imaging in a way that is uniquely tractable in zebrafish (Chang et al. 2012; Early et al. 2018; Oprişoreanu et al. 2021).

In this section, we highlight the tools used in zebrafish for the imaging of brain structure and neuronal substructure, the tracking of neuronal activity during behaviors, and the manipulation of neuronal circuits to probe causality, with an emphasis on their utility in the study of neurodegenerative disease models.

#### Structural imaging—whole brain, single neuron, and subcellular

The zebrafish brain is easy to image for gross alterations in the organization of brain anatomy, region-specific changes in volume, and loss or expansion of specific cell types. These approaches are greatly facilitated by large collections of transgenic lines and validated antisense probes that label discrete populations of neurons and glia, which are deposited as highly annotated expression maps in community resources like MapZebrain and other larval zebrafish brain atlases (Table 2) (Randlett et al. 2015; Kunst et al. 2019). Brain imaging data from genetic or experimental manipulations can be aligned to reference brain images and used to query these databases to identify gene expression or structural changes. Early versions of this approach in neurodegeneration studies used live imaging to observe neuronal degeneration in a tauopathy model (Paquet et al. 2009) and histaminergic neuron loss in a *psen1* loss-of-function mutant (Sundvik et al. 2013). Taking full advantage of reference brain mapping, a more recent example was able to uncover subtle but consistent altered serotonin signaling components in just some, but not all, brain areas in mutants lacking the AD risk gene *sorl1* (Kroll et al. 2025).

Important sub-cellular structure is also straightforward to image in live zebrafish, with transgenic tools available to visualize dendrites, axons, and synapses. Methods such as in vivo electroporation or photoconvertible GFP can be used to sparsely label single cells for clear tracing of neuronal projections (Hatta et al. 2006; Sato et al. 2006; Barsh et al. 2017). A variety of tools have also been developed to observe pre- and post-synapses dynamically in vivo. Transgenic expression of synaptophysin-GFP has been used to observe pre-synaptic development and plasticity in circuits ranging from retinal ganglion cells (Du et al. 2018) to wake-regulating orexin/hypocretin neurons in the hypothalamus (Elbaz et al. 2017). The postsynaptic site of synapses can be non-invasively imaged using Fibronectin intrabodies generated by mRNA display (FingRs) that tag scaffold proteins of either excitatory (PSD-95) or inhibitory (Gephryin) synapses (Gross et al. 2013). These tools have been used to track synapse formation and elimination over long timescales (Son et al. 2016) in the context of both learning and memory (Dempsey et al. 2022) and sleep (Suppermpool et al. 2024). Given that sleep and memory processes are profoundly disrupted in neurodegenerative disease, applying these tools to disease models remains an underexplored but promising strategy. For instance, zebrafish overexpressing human tau isoforms associated with AD exhibit synapse loss alongside neurodegeneration (Bai et al. 2024); in vivo imaging with FinGR-based synapse labeling tools could help map the spatial and temporal trajectory of these changes at a brain-wide scale.

#### Neuronal activity snapshots

Although sophisticated methods to observe real-time neuronal activity have been developed, the fairly low throughput of live imaging means that many applications still rely on single time-point snapshots of indirect markers of neuronal activity. Common methods, which have the advantage of being performed in many brains in parallel, include in situ hybridization or HCR staining for the immediate early gene *c-fos* to capture larger, sustained increases in neuronal activity (Sagar et al. 1988) and antibody labeling of phospho-ERK (p-ERK) to observe more rapid changes in neuronal activity (Randlett et al. 2015). These approaches are particularly useful for assessing activity patterns across the whole brain, within genetically defined circuits, or across mutant lines with altered behavioral phenotypes. For example, Ozcan et al. (2020) used both *c-fos* and p-ERK mapping to examine the impact of different amyloid-β (Aβ) oligomer species on brain activity, finding that shorter forms induced strong activation in wake-related brain regions, while longer oligomeric forms suppressed neuronal activity globally.

#### Genetically encoded calcium, voltage, and neuromodulatory imaging

Zebrafish neuroscience has come to heavily rely on genetically encoded calcium indicators (GECIs) to monitor neuronal activity with high spatiotemporal resolution in vivo. One of the most widely used GECIs is GCaMP, which fluoresces upon calcium binding to provide a dynamic readout of neuronal firing. GCaMP-based imaging has been used to map whole-brain activity during diverse normal and diseased behavioral states, including sensorimotor processing, sleep, and seizures (Muto and Kawakami 2011; Ahrens et al. 2013; Naumann et al. 2016; Turrini et al. 2017). However, most implementations of calcium imaging require immobilizing larvae in agarose or with paralytics (Ahrens et al. 2013; Turrini et al. 2017), which limits the phenotyping throughput and the types of behaviors that can be tested, although even complex behaviors like hunting are still performed in head fixed, tail-free preparations (Hasani et al. 2023). More recently, the development of tracking microscopy that can record whole-brain calcium imaging data from freely swimming larvae (Robson and Li 2024), promises to expand the possible applications even further, as in the recent demonstration of place cells in the larval zebrafish telencephalon (Yang et al. 2024).

Although GECIs reliably report on neuronal activity and can be used in many contexts to infer spiking activity, the kinetics of intracellular calcium is too slow to capture the very rapid changes in membrane potential that can occur at millisecond timescales. Genetically encoded voltage indicators (GEVIs), which fluoresce in response to changes in membrane potential, have been successfully used in zebrafish for the direct visualization of electrical activity in neurons in the early embryo (Silic and Zhang 2018), cerebellum, and spinal cord (Miyazawa et al. 2018; Hiyoshi et al. 2021).

Additional genetically encoded sensors for neurotransmitters and neuromodulators have been developed and used for imaging neuronal signaling dynamics in zebrafish brains. GRAB sensors (G-protein-coupled receptor activation-based sensors) for noradrenaline (GRAB-NE), adenosine (GRAB-Ado), serotonin (GRAB-5HT), dopamine (GRAB-DA), and extracellular ATP (GRAB-ATP) report in real time the dynamics of these key neuromodulators (Sun et al. 2018; Feng et al. 2019; Jing et al. 2019; Wu et al. 2023; Deng et al. 2024). For example, GRAB-NE was used to track NE release in the zebrafish midbrain in response to looming stimuli to show how NE sculpts the larvae's sensitivity to induce escape responses (Feng et al. 2019). Tracking these signaling events in neurodegenerative disease models will increase the precision of determining changes in brain-wide neuronal signaling. For example, GRAB-DA sensors could be applied in zebrafish PD models to track progression of dopaminergic signaling dysfunction in distinct brain areas (Sun et al. 2018).

**Fig. 3.**
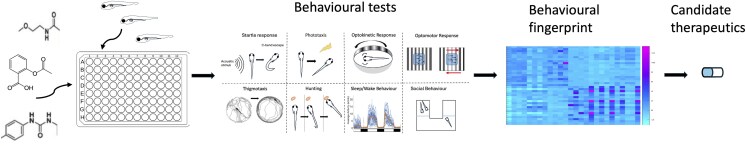
High-throughput behavioral pharmacology. Hundreds of drugs can be tested across multiple genotypes and phenotypes in parallel to accelerate the discovery of candidate therapeutic targets.

Although in vivo imaging of neuronal and neuromodulatory activity has yet to be widely adopted in zebrafish studies of neurodegeneration, these approaches hold considerable promise to directly link molecular pathology to circuit-level dysfunction. For example, GCaMP imaging could reveal neurodegenerative disease-associated vulnerabilities in network excitability or sensory processing. Within a zebrafish phenotyping pipeline (Fig. 3), these tools could complement higher-throughput behavioral assays and static molecular readouts by enabling targeted, lower-throughput imaging studies in defined brain regions or behaviors, thereby providing a bridge from large-scale screening to mechanistic dissection of disease-related circuit dysfunction.

#### Circuit manipulations in behaving fish

Zebrafish are highly amenable to circuit manipulations that reveal causal links between neural activity and behavior. These methods, which test for the necessity and sufficiency of neuronal populations for controlling a given behavior, range from irreversible, targeted ablations, to reversible strategies like optogenetics and chemogenetics. When combined with neuronal imaging and behavioral analysis, direct manipulation of neurons can test the functional roles of specific circuits and evaluate whether this control is disordered in models of disease.

Although irreversible and limited to loss of function, neuronal ablations remain a straightforward way to test the importance of neurons for given behavioral outputs. Direct laser ablation with multiphoton microscopy allows extremely precise deletion of even single neurons and has been used to dissect the roles of neurons in many complex behavioral contexts, including prey capture (Roeser and Baier 2003; Muto and Kawakami 2018). Higher throughput methods use genetically encoded, drug or light-inducible toxins to selectively eliminate whole classes of neurons using transgenic lines to drive the toxin expression. Common genetically encoded approaches to ablation include the nitroreductase system, which enzymatically converts the prodrug metronidazole into a cytotoxic metabolite that kills targeted cells and light-inducible effectors such as KillerRed, which generate reactive oxygen species upon illumination, leading to oxidative damage and cell ablation (White and Mumm 2013; Tabor et al. 2014; Buckley et al. 2017). These methods are particularly useful for mimicking selective cell loss seen in neurodegenerative diseases, for example, using the nitroreductase system to make targeted ablations of dopamine neurons to model PD (Kim et al. 2021).

Optogenetics enables reversible and precise control of neuronal activity and has become a widely adopted technique in zebrafish neuroscience (Portugues et al. 2013). Common optogenetic tools, including channelrhodopsins for depolarization and anion channelrhodopsins (GtACR1), halorhodopsin (eNpHR), and archaerhodopsins (ArchT) for hyperpolarization (Antinucci et al. 2020) have been used extensively to dissect sensorimotor transformations, map functional connectivity, and control behavior in both freely swimming and head-fixed larvae. For example, optogenetic activation has been used to probe the serotonergic modulation of vigilance states (Oikonomou et al. 2019; Zhao et al. 2024), while silencing of interneurons helped define circuit logic in locomotor and visual pathways (Arrenberg et al. 2009; Wyart et al. 2009).

As an alternative to optogenetics, chemogenetic methods control neuronal excitability and activity using engineered receptors or channels that are activated by synthetic ligands and allow for less-invasive and longer-term modulation of neuronal excitability, leading to more naturalistic patterns of neuronal firing. In particular, TRP channels activated by lower doses of capsaicin, which zebrafish do not naturally sense, have been employed to drive rapid, reversible activation of targeted neurons in behaving zebrafish larvae (S. Chen et al. 2016). For example, TRPV1/capsaicin chemogenetics was used to show that cerebellar Purkinje cells encode tilt direction and are essential for maintaining postural balance along the pitch axis (Auer et al. 2025).

While initially developed for circuit mapping, optogenetic and chemogenetic approaches now hold considerable promise for modeling and interrogating neurodegenerative disease mechanisms. These tools could be used to test causal hypotheses, such as whether neuronal hyperexcitability accelerates cell loss, whether restoring activity in degenerating circuits is protective, or how neuromodulatory pathways (eg cholinergic, noradrenergic) influence resilience. Within a broader experimental pipeline, circuit manipulation could complement high-throughput behavioral phenotyping and static molecular readouts by allowing mechanistic follow-up in defined brain regions or cell types identified as vulnerable to neurodegeneration, providing a dynamic link between molecular pathology, circuit dysfunction, and behavioral outcomes.

### Small molecule screening and behavioral clustering

Zebrafish are a versatile system for small molecule screening, with unique advantages over cell-based and invertebrate models. One major strength is their ability to absorb compounds directly from the water, eliminating the need for injection-based delivery. In early larval stages, an incomplete blood–brain barrier even facilitates the penetration of many molecules into the brain (Fleming et al. 2013). Zebrafish drug experiments capture complex pharmacokinetic considerations, including absorption, distribution, metabolism, excretion, and toxicity (ADME-Tox), which make the model highly suitable for both toxicology and functional studies (Jeong et al. 2008). The ease of testing in multi-well plates allows for high-throughput analyses using robotic drug delivery systems and automated videography or microscopy (Chang et al. 2012). Zebrafish small molecule screens have investigated a variety of biological phenomena, including organophosphate toxins (Jin et al. 2013), epilepsy (Baraban et al. 2013), and sleep (Rihel et al. 2010b).

Zebrafish larvae facilitate the combination of high-throughput behavioral analysis and drug screening (Fig. 3). Both stimulus-driven and complex behaviors, from acoustic startle to circadian rhythms (see *Behavioral Phenotyping* section), can be uniformly conducted simultaneously across many wells that contain different pharmacological treatments (Rihel et al. 2010b; Pelkowski et al. 2011; Bruni et al. 2016; Mosser et al. 2019). The quantifiability and simplicity of these behavioral assays support multi-parameter phenotyping approaches like behavioral fingerprinting, which cluster chemical compounds or mutants based on their multidimensional activity profiles to link treatment or genetic groups to specific molecular mechanisms (Kokel et al. 2010; Rihel et al. 2010b; Bruni et al. 2016). Behavioral fingerprinting has been used to reveal new functional compound classes, including previously uncharacterized monoamine oxidases (Kokel et al. 2010; Rihel et al. 2010b), uncovered neurotransmitter signaling disrupted in genetic mutants, and predicted small molecules capable of ameliorating mutant phenotypes (Kokel et al. 2010; Hoffman et al. 2016; Kroll et al. 2025). For instance, small-molecule induced changes in zebrafish locomotor activity across multiple days linked new signaling pathways to sleep (Rihel et al. 2010b), and behavioral profiling of the zebrafish *cntnap2* mutant model of autism successfully predicted estrogen-related compounds that re-normalized their nighttime hyperactive behavior (Hoffman et al. 2016).

Next generation approaches go beyond behavior by combining small molecule screening with high-throughput whole-brain GCaMP imaging. Using unsupervised clustering algorithms to group compounds by their effects on global neuronal dynamics, one study was able to identify specific drug combinations that restore network function in a Dravet syndrome model (Ghannad-Rezaie et al. 2019). These polytherapies correct abnormal network connectivity and cut spontaneous seizures at lower doses than single drug treatments and were discovered by avoiding brute-force combination screens (Ghannad-Rezaie et al. 2019). This system-level study illustrates how whole-brain responses to drugs can be used to triage neuroactive compounds to quickly make unexpected links between genetic pathways, neuronal circuits, and small molecules.

In the context of neurodegenerative disease, more targeted and rationally-designed small molecule screening approaches in zebrafish have already proven successful, for example by identifying compounds that reduce tau hyperphosphorylation in a transgenic huTAU-P301L model (Paquet et al. 2009) or that prevent acute Amyloid-beta-induced changes in sleep patterns (Ozcan et al. 2020). In another example, one small screen of 17 drugs was able to confirm that riluzole reduced neuronal stress in a *sod1* mutant model of ALS (Mcgown et al. 2016). Larger-scale drug repurposing screens have also been successful in identifying promising therapeutic lead compounds. One chemical screen in a zebrafish model of rod-photoreceptor degeneration (the *rho:eGFP-tau-WT* model), found that carbonic anhydrase inhibitors could prevent rod degeneration, a finding that was validated in mice using a related, clinically approved glaucoma medication (Lopez et al. 2025). Similarly, a small molecule screen in a dopamine neuron ablation model of PD found that renin–angiotensin–aldosterone system inhibitors, which are already clinically approved for cardiovascular conditions, could protect against neurodegeneration (Kim et al. 2021). In the future, combining small molecule screening with behavioral profiling will be an effective way to accelerate drug discovery, as in one recent study that used a behavioral fingerprinting analysis of 900 drug-induced zebrafish phenotypes to quickly find 64 compounds that mimicked cyclosporin A, a drug with promising potential in the treatment of AD (Del Rosario Hernandez et al. 2024).

Looking ahead, zebrafish chemical screening is poised to move beyond simple hit identification toward integrated, systems-level discovery that will combine behavioral profiling with mutant libraries, transcriptomic readouts, and brain-wide imaging to generate multidimensional, multi-tiered phenotypic signatures. Such data-rich approaches will enhance compound clustering, facilitate target deconvolution, and identify drug combinations that target complementary aspects of disease pathology. Advances in computational clustering, machine learning, and drug–phenotype databases will further enable these integrated screens and position zebrafish at the forefront of neurodegenerative drug discovery.

## Conclusion

Zebrafish offer unique advantages for dissecting neurodegenerative disease mechanisms, combining optical transparency, genetic accessibility, and high-throughput phenotyping in an intact, living system. These pluses enable a streamlined pipeline in which neurodegenerative disease models systematically undergo transcriptomic profiling, behavioral phenotyping, whole-brain imaging, and pharmacological manipulations, in parallel and at scale. Although neurodegenerative diseases typically manifest later in life, it is not paradoxical to investigate these disorders in larval zebrafish stages because the systems-level framework outlined here can identify early circuit-level and molecular vulnerabilities linked to disease genes and facilitate the search for candidate therapeutic interventions. The zebrafish model offers a powerful framework for finding early dysfunctions in cell signaling or behavioral circuits that arise before the onset of overt disease pathology, the discovery of which will be essential for combating neurodegenerative conditions.
